# Understanding aneuploidy in cancer through the lens of system inheritance, fuzzy inheritance and emergence of new genome systems

**DOI:** 10.1186/s13039-018-0376-2

**Published:** 2018-05-10

**Authors:** Christine J. Ye, Sarah Regan, Guo Liu, Sarah Alemara, Henry H. Heng

**Affiliations:** 1 The Division of Hematology/Oncology, Department of Internal Medicine, University of Michigan, Ann Arbor, MI 48109 USA; 2 Center for Molecular Medicine and Genomics, Wayne State University School of Medicine, Detroit, MI 48201 USA; 3 Department of Pathology, Wayne State University School of Medicine, Detroit, MI 48201 USA

**Keywords:** Adaptive system, Aneuploidy, Cancer evolution, Complexity, Emergence of new genome, Fuzzy inheritance, Genome theory, Non-clonal chromosome aberrations (NCCAs), Punctuated evolution, System inheritance

## Abstract

**Background:**

In the past 15 years, impressive progress has been made to understand the molecular mechanism behind aneuploidy, largely due to the effort of using various -omics approaches to study model systems (e.g. yeast and mouse models) and patient samples, as well as the new realization that chromosome alteration-mediated genome instability plays the key role in cancer. As the molecular characterization of the causes and effects of aneuploidy progresses, the search for the general mechanism of how aneuploidy contributes to cancer becomes increasingly challenging: since aneuploidy can be linked to diverse molecular pathways (in regards to both cause and effect), the chances of it being cancerous is highly context-dependent, making it more difficult to study than individual molecular mechanisms. When so many genomic and environmental factors can be linked to aneuploidy, and most of them not commonly shared among patients, the practical value of characterizing additional genetic/epigenetic factors contributing to aneuploidy decreases.

**Results:**

Based on the fact that cancer typically represents a complex adaptive system, where there is no linear relationship between lower-level agents (such as each individual gene mutation) and emergent properties (such as cancer phenotypes), we call for a new strategy based on the evolutionary mechanism of aneuploidy in cancer, rather than continuous analysis of various individual molecular mechanisms. To illustrate our viewpoint, we have briefly reviewed both the progress and challenges in this field, suggesting the incorporation of an evolutionary-based mechanism to unify diverse molecular mechanisms. To further clarify this rationale, we will discuss some key concepts of the genome theory of cancer evolution, including system inheritance, fuzzy inheritance, and cancer as a newly emergent cellular system.

**Conclusion:**

Illustrating how aneuploidy impacts system inheritance, fuzzy inheritance and the emergence of new systems is of great importance. Such synthesis encourages efforts to apply the principles/approaches of complex adaptive systems to ultimately understand aneuploidy in cancer.

## Background and progress

Why is aneuploidy commonly observed in various cancer types? How does aneuploidy directly or indirectly contribute to cancer? Is aneuploidy good or bad for cancer initiation and progression, and how does it affect treatment response? What is the relationship between aneuploidy and other genetic/epigenetic aberrations? How important is it to study each individual molecular mechanism that can be linked to aneuploidy? What are the general mechanisms (cause and effect) for generating aneuploidy? Why can aneuploidy be detected from other diseases? And what is the biological significance of aneuploidy in normal tissues for normal individuals? … These questions represent some long-debated issues in the field of cancer research, ever since Theodor Boveri recognized the link between aneuploidy and cancer over a century ago [1–4].

Specific aneuploidy has been observed in various non-cancer diseases: Down syndrome with trisomy chromosome 21, Edwards syndrome with trisomy 18, Patau syndrome with trisomy 13, Klinefelter’s syndrome with an extra X, and Turner’s syndrome with the absence of one X. While clonal aneuploidy is also detected in some cancers, such as chronic lymphocytic leukemia (CLL) with trisomy 12 and acute myeloid leukemia (AML) with trisomy 8, the percentage of such cancer patients with the signature clonal aneuploidy is much lower (18% for CLL and 8.5% for AML) compared to those with Down syndrome (over 95% of all patients), suggesting that there are more diverse genomic factors contributing to cancer (even for the liquid cancer type) than those non-cancer genetic diseases.

Altogether, the complexity of aneuploidy makes studying its relationship with cancer extremely challenging (Table 1). Some known complications include: a) most cancer cases display non-clonal aneuploidy (impeding the fact that clonal aneuploidy has been much more commonly researched for decades) [5–9], b) aneuploidy often occurs in combination with other types of genetic/epigenetic and genomic aberrations (translocations and polyploidy) (Table 2) c) there is often a variable degree of somatic mosaicism [10–13], and d) there is a complex, dynamic relationship between aneuploidy and genome instability (Table 3). Interestingly, many common and complex diseases have been linked to non-clonal aneuploidy and somatic mosaicism as well [14, 15], which has led to efforts to search for commonly shared mechanisms among different diseases or illness conditions [16–19]. It is worth noting that aneuploidy can also be detected from the normal developmental process [20–22].

**Table 1:** Explanations of key terminologies

**Aneuploidy** is a changed genomic state with an abnormal number of chromosomes in a cell. In cancer, most aneuploidy is not clonal or constitutional. Recently, a looser definition of aneuploidy has been used to analyze DNA sequence data, which includes partial chromosomal changes and somatic copy number aberration (SCNA). Such usage is not precise, as germline CNVs and SCNA represents the variable copy number of specific sequences, which is not the same as the entire abnormal chromosome(s). According to the genome theory, the chromosome represents a coding system, so the impact of aneuploidy is therefore much more significant than SCNA. The mechanisms causing somatic aneuploidy are many; examples can be found in Table 2.	
**CIN:** Chromosome instability (CIN) refers the rate (cell-to-cell variability) of changed karyotypes of a given cell population. There are two types of CIN: numerical and structural. Numerical CIN is determined by the gain or loss of whole chromosomes or fractions of chromosomes (aneuploidy), as well as other forms. Structural CIN, on the other hand, is determined by structural NCCAs. Numerical and structural CIN often co-exist. CIN can be effectively measured by the frequency and type of NCCAs.	
**Type I and type II CIN:** CIN can be classified into two types based on its involved molecular mechanisms. Type I CIN is directly linked to the maintenance of genome integrity within the chromosomal cycle, including the chromosomal machinery, checkpoints, and repair systems (see Table 3). Type I CIN is often detected in chromosome instability syndromes which provide good examples of direct “molecular causative relationship” between identified genes and CIN. However, mutations to type I genes are rare and they do not explain sporadic cancer. In contrast, the mechanisms of type II CIN are often associated with non-genetic factors such as the micro-environment and physiological processes, which do not have a direct molecular causative explanation. The diverse type II mechanisms all share one common feature: they are involved in the cellular system’s response to stress, increasing heritable changes [50].	
**Fuzzy inheritance:** In contrast to the gene theory, which states that a gene codes for a specific, fixed phenotype, the genome theory suggests that most genes code for a range of potential phenotypes. From this “fuzzy” range of phenotypes, the respective environment can then allow the best-suited status to be “chosen” [4, 37, 59]. For example, the gene for pea color codes for an entire potential spectrum of colors, from yellow to intense green (including blends of yellow and green, or green with yellow spots), not just two fixed, distinctive colors (yellow or intense green). In cancer, the emergence of “genomic context” adds yet another layer of complexity and instability that pushes fuzzy inheritance’s dynamics to a maximal status.	
**Macro-and micro-cellular evolution:** Macro-cellular evolution refers to karyotype change-mediated somatic cell evolution, which alters the genome context of a given cellular system. In contrast, micro-cellular evolution refers to gene/epigene change-mediated evolution, which modifies a given cellular system within the same karyotype. Macroevolution and microevolution respectively refer to organismal evolution at the above-species level and at the population level within a species.	
**System inheritance:** Unlike the gene-defined “parts inheritance” (the instructions for making a given protein or RNA), a new three-dimensional genomic topologic coding, or the blueprint of the genome, is defined by the order of genes or other DNA sequences along and among the chromosomes of a given genome. This blueprint encodes how genes interact as an emergent property, which provides the instructions for how genomic networks work [4, 37, 66].

**Table 2:** Examples of different types of causative factors of aneuploidy

1. Gene mutations/epigenetic alterations	[111–113]
Mitotic checkpoint defects, e.g. *BUB1*, *MAD1* and *CENPE*	[114, 115]
Microtubule attachment defects, e.g. *aurora kinase B, Cydlin A*,	
Mitotic spindle and centrosome defects	[116]
Other CIN-related mutation, e.g. *p53, ATM*	[117]
2. Stress- (physiological, pathological and pharmaceutical) related responses	
Defective mitotic figures (condensation defects) (DMF, sticky chromosomes)	[50, 78, 106]
Chromosome fragmentations (C-Frags)	[115, 116]
Genome chaos	[5, 6, 37, 59, 67]
Chromosomal cycle variations (replication, condensation, segregation, de-condensation)	[104]
Non-specific stress (triggers type II CIN)	[50]
3. Genome system variability	
Fuzzy inheritance	[4, 37, 59]
Cellular adaptation	[37, 95]
Survival under high stress	[56, 67]

To illustrate the viewpoint that many genomic and environmental factors can contribute to aneuploidy, a few examples are presented, among a large number of publications. We focus more on the examples that feature a cytogenetic perspective, as these are currently less popular compared to gene mutation studies, despite their importance

**Table 3:** Examples of interesting observations in aneuploidy studies including some conflicting data.  Some comments are also offered to explain them

1. The dynamic relationship between aneuploidy and CIN Aneuploidy generates CIN, including increased chromosome loss, mutation rate and defective DNA damage repair [39, 119]. The relationship between aneuploidy and CIN can be envisioned as a “vicious cycle,” wherein one potentiates the other [120]. The “stress–CIN–cancer evolution relationship” can also be used to discuss the relationship between aneuploidy and cancer [50]. Elevated transcriptome dynamics are linked to karyotype changes which impact multiple genetic/epigenetic interactions [121–123] Aneuploidy is less influential compared to structure alterations [54]. CIN rates might be more predictive for tumor outcome than assessing aneuploidy rates alone [54, 124]. Many cancer cell lines with aneuploidy are relatively stable (an example of fuzzy inheritance of some relatively stable systems) [37]. Genome chaos, including karyoplast budding, giant cells and mitotic catastrophe, is often associated with aneuploidy [67, 125–127]. Chromosomal condensation defects (DMFs) and Chromosome fragmentation (C-Frags) can generate aneuploidy [37, 106, 118]. Aneuploidy (in the form of mosaicism) represents a common phenomenon. We may all have a touch of Down syndrome [128, 129]. Aneuploidy is a main feature among individual cancer cell lines. The rate of aneuploidy seems inherited [72]. Genomic *PTEN* deletion size influences the landscape of aneuploidy and outcome in prostate cancer [130]. ATM and p21 cooperate to suppress aneuploidy and tumor development [117]	
2. The complex relationship between aneuploidy and immune response When co-cultured with natural killer cells, aneuploidy cells with complex karyotype-induced senescent cells were selectively cleared [131]. High copy number alterations in melanoma patients are linked with less effective response to immune checkpoint blockade anti–CTLA-4 [52].	
3. Biological impact of aneuploidy Aneuploidy changes the genomic coding, which affects the transcriptome, proteome, network structure, incidence of CIN and phenotypes [4, 37, 132]. Chromosome mis-segregation per se can alter the genome in many ways in addition to chromosome gain or loss [133]. Aneuploidy puts pressure on the protein machinery and quality control, which generates a global stress response, reducing cell proliferation [133]. Both specific gene effects and the typical aneuploidy stress response contribute to new genomic coding or/and increased system stress, which can impact the emergent process of cancer evolution ([133], current paper) Karyotype status (e.g. aneuploidy and polyploidy) can restore functions of specific genes (e.g. *MYO1*). Thus, genomic coding changes gene coding [83]. The chromosomal size involved in aneuploidy is inversely correlated to the resulting fitness [134]. The risk of cancers to metastasize is proportional to the degree of cancer-specific aneuploidy [48]. There is a dynamic relationship between epigenetic events and aneuploidy; epigenetic marks play a role in the control of chromosome segregation and integrity; aneuploidy impacts chromatin silencing [135–137]. New approaches are needed to study the complexity of systems, including that of aneuploidy-mediated karyotype evolution [138, 94, 110].	

Such complexity did however discourage aneuploidy research, as cloning and characterizing individual cancer genes had promised much more certainty. During the peak era of oncogene- and tumor suppressor gene-focused research, for example, the importance of aneuploidy was largely ignored, due to high expectations from the gene mutation theory of cancer. As a result, efforts to systematically study aneuploidy in cancer, especially based on the belief that aneuploidy is much more important than gene mutations, are limited to a small number of research groups [23–26]. One of the popular viewpoints was that cancer gene mutations hold the key to understanding cancer, whereas chromosomes were just vehicles of genes; it was furthermore argued that most chromosomal changes are either incidental or the consequence of gene mutations.

While it was observed that some chromosomes display a tumor suppressor function following cell/chromosome fusion experiments [27], efforts were focused on cloning tumor suppressor genes [28]. The lack of easy-to-recognize patterns in aneuploidy has certainly reduced the enthusiasm of most funding agents about this topic, especially when gene mutation research has promised to identify the key common gene mutations for cancer.

One important publication has classified cancer into two major types based on observed molecular mechanisms: chromosome instability (CIN) and microsatellite instability [29]. Remarkably, the majority of colon cancers display CIN. The fact that most cancers can be linked to chromosomal instability was a surprise to many who primarily study cancer genes.

If the majority of cancers are linked to CIN, and aneuploidy contributes to CIN, more attention needs to be paid to aneuploidy [30]. Based on this concept, increased efforts were focused on identifying genes that are responsible for aneuploidy. Many individual genes and molecular pathways involving chromosomal machinery/integrity have been linked to aneuploidy. For example, a list of identified genes that contribute to aneuploidy-mediated cancer includes germline BUBR1 mutation, which leads to aneuploidy and cancer predisposition [31]. Additional examples can be found in Table 2.

Another important factor that promotes aneuploidy research is the popularization of copy number variation studies of the human genome [32–34]. If various individual instances of CNV are of importance, large scale CNVs caused by aneuploidy should be too, despite the fact that the search for specific genes related to aneuploidy (such as chromosome 21) have traditionally been the main focus. The availability of various technologies that can detect CNV have now revolutionized molecular cytogenetics. It should be mentioned that the cytogenetically visible copy number variations (CG-CNVs) need more attention [35]. Regarding the framework of fuzzy inheritance, CNVs, CG-CNVs, small supernumerary marker chromosomes and aneuploidy represent different degrees of fuzziness, which are likely reflected by quantitative difference or combinational effect. It is important to integrate these with analyses of system emergence [4, 36, 37].

In recent years, due in part to the disappointment that has come from attempting to identify the common driver gene mutation, and more significantly, due to the realization that genome instability plays an important role in cancer, aneuploidy studies have gained momentum. In particular, the popularity of studying aneuploidy in cancer has been promoted by some yeast biologists. Taking advantage of yeast model systems, they have applied cutting-edge molecular and genomic technologies to illustrate the molecular mechanisms that link aneuploidy to biological functions [38–42]; by translating their discoveries into cancer research, they have brought the spotlight on aneuploidy research in cancer [43, 44] (Tables 2, 3). Interestingly, a complex relationship between aneuploidy and cancer has also been revealed, proposing that aneuploidy can either promote or inhibit cancer progression depending on the evolutionary context. This has led to the paradox of aneuploidy in cancer [45, 46].

There have been attitude changes towards the study of aneuploidy as well. When direct evidence simultaneously characterized gene mutation and chromosomal aberrations as drivers for the phenotypic implication of metastasis [47], the authors clearly emphasized CIN, and the potentially involved gene was not even mentioned in the title. This likely represents a new favored approach focusing on genome-level changes. There is also the realization that chromosomal aberrations contribute more significantly to metastasis than gene mutations do [48] which supports the hypothesis that chromosomal aberration-mediated genome evolution is responsible for all major transitions in cancer evolution, including metastasis and drug resistance [49, 50]. Furthermore, and surprisingly to many molecular researchers, chromosome aberration profiles have been demonstrated to have a much stronger prediction value in the clinic compared to DNA sequencing profiles [51]. This conclusion has gained strong support from various cancer genome sequencing projects [52, 53], which prompts an important question regarding the differential contribution of chromosome aberrations and gene mutations to the cancer genotype. All together, rapidly accumulated data has forcefully highlighted the importance of aneuploidy in current cancer research, and more detailed molecular information linking individual gene mutations or epigenetic events to aneuploidy will soon flourish.

### Challenges for predicting cancer status based solely on the molecular mechanisms of aneuploidy

Like other hallmarks of cancer, aneuploidy has now become a hot topic. A predictable new trend is that more researchers will join the effort to link all possible genetic/epigenetic and environmental factors to aneuploidy and cancer. However, as we have extensively discussed, due to biocomplexity (i.e. that many individual factors can contribute to the same phenotype), it is possible that merely collecting more diverse molecular data linking gene mutation and environmental factors to aneuploidy is not the best way to advance this field. This is because there will be too many factors involved, most of them lacking the power to predict cancer status [54, 55].

This viewpoint has been articulated by the evolutionary mechanism of cancer and its relationship with individual molecular mechanisms [50, 56]. In brief, cancer evolution can be understood by the dynamic interaction among four key components: internal and external stress; elevated genetic and non-genetic variations (either necessary for cellular adaptation or resulting from cellular damages under stress); genome-based macro-cellular evolution (genome replacement, emergent as new systems); and multiple levels of system constraint which prevent/slow down cancer evolution (from tissue/organ organization to the immune system and mind-body interaction). Since the sources of stress are unlimited and unavoidable (as they are required by all living systems), there are large numbers of gene mutations/epigenetic events/chromosomal aberrations, such as aneuploidy, that can be linked to stress-mediated genomic variants; furthermore, as environmental constraints are constantly changing, even identical instances of aneuploidy will have completely different outcomes in the context of cancer evolution, as the results of each independent run of evolution will most likely differ. Solely knowing the mechanism of aneuploidy limits the predicting power for cancer. Furthermore, hundreds of gene mutations can contribute to aneuploidy, and the various contexts of cancer evolution are almost unlimited. Based on this rationale, we promote the idea of using the evolutionary mechanism of cancer to unify diverse individual molecular mechanisms of cancer (4).

Unfortunately, such ideas have received little attention within the cancer research community, due in part to the traditional molecular characterization of gene mutations, and possibly more so due to many cancer biologists’ unfamiliarity with complexity science and a lack of understanding of the key principles of bio-emergence. It is thus necessary to discuss this issue of aneuploidy in cancer using the framework of the complex adaptive system [37].

A complex adaptive system is a system made up of many individual parts (agents) with nonlinear dynamical interaction. Due to the key emergent relationship between the lower level of heterogeneous agents and the behavior of the entire system, a detailed understanding of the individual parts does not automatically convey a determinist’s understanding of the whole system’s behavior. There are no fixed, dominant agents within the adaptive system, and when agents of the system are changed, the system adapts or reacts. Moreover, small changes in initial conditions can generate large changes in the system’s outcome, and stochasticity is also frequently involved [57, 58]. As a result, the reductionist approaches which have triumphed in molecular biology may be fundamentally limiting when attempting to understand complex adaptive systems.

Cancer is typically a complex adaptive system involving multiple levels of agent interactions and genotype/phenotype emergence among different types of tissue/organ constraints. In such a system, aneuploidy represents only one type of agent, despite its importance. There is a complex interaction among different levels of genetic organization, which involves phase transitions among clonal and non-clonal cellular populations, and the final emergence of different genome-defined cellular systems under highly dynamic cellular environments and the process of cancer evolution. This reality of cancer evolution explains why it is so challenging to predict the final phenotype based on an understanding of one type of agent. The take-home message is that simply understanding the molecular mechanism (both cause and effect) of aneuploidy is far from enough. A better strategy is to monitor the evolutionary process by measuring evolutionary potential. For example, the overall degree of CIN is more predictive than individual gene mutation profile [54]; large-scale chromosomal structural aberrations can often have a more profound impact on cancer evolution (even though aneuploidy often leads to structural aberrations as well); and the landscape of chromosomal aberrations is more predictive than gene mutation landscapes. Furthermore, the initial factor and the evolutionary trajectory differ in complex systems. It is now accepted that treatment options can often drastically and rapidly change the genetic landscape of the cancer [59].

In addition to the challenge that cancer is a complex adaptive system, it should be understood that current molecular knowledge of aneuploidy is mainly derived from model systems, which can differ from cancer systems in patients. The following limitations are briefly mentioned to bring the reader’s attention to them, and they are also useful for explaining some conflicting observations. First, the platform of the yeast model differs from human cellular populations within tissue. Different species display the feature of aneuploidy quite variably. In budding yeast *Saccharomyces cerevisiae*, aneuploidy is not uncommon and exists in natural populations; in plants, organisms can tolerate whole chromosome aneuploidy without triggering CIN; in mice, every single whole chromosome gain or loss is embryonic lethal [60]; in humans, the situation is similar to that of mice, with the exception of a few chromosome gains such as 13, 18 and 21. The pattern of evolution also differs when diverse types of cellular selection are involved, in addition to differing types of system constraints. For cancer evolution in reality, the overall complexity and the level of dynamics is much higher, which can often change the game completely. In the future, multiple cellular models might be helpful to certain degrees, especially when the time variable (i.e. development and aging) is added in to the equation.

Second, the status of clonal and non-clonal aneuploidy differs between many model systems and the reality of cancer. So far, for many yeast and human cell models, aneuploidy stains are created with clonal populations in which most cells display the same extra chromosomes. In contrast, for many solid tumors, aneuploidy exists in non-clonal forms. Such differences may contribute to some misperceptions, thus requiring further studies. For example, the analysis of trisomic cells from human patients with congenital aneuploidy syndromes did not display any increased CIN, concluding that aneuploidy itself does not lead to cancer-like CIN [61]. We have mentioned the significant difference between constitutional aneuploidy and the acquired aneuploidy observed in cancers. Constitutional aneuploidy is a clonal-chromosome aberration (CCA), whereas many acquired somatic aneuploidies are nonclonal-chromosome aberrations (NCCAs). In the cellular environment of trisomy 21, trisomy 21 is the dominating “normal” genome, and any other genomes (including the “normal” 46 XY or XX karyotype) are relatively “abnormal;” the homeostasis of trisomy 21 could actually generate less cellular variation, which explains the resulting low levels of cell-to-cell variations. Based on this analysis, we suggested that although specific constitutional aneuploidy alone is not sufficient for generating numerical CIN, it is necessary to examine the impact of non-recurrent, stochastic aneuploidy on generating all types of CIN [62].

Third, many models feature simple types of aneuploidy (with one extra chromosome within an otherwise normal karyotype, for example), which is easier to analyze with repeatable results. In contrast, in the setting of cancer evolution, aneuploidy is often coupled with structural chromosomal changes and/or polyploidy. In addition, the rate of aneuploidy within the population is often lower than in clonal populations of model systems, while for each cell with aneuploidy, the heterogeneity is higher than cells from model systems (there are often multiple extra chromosomes, for example). Such differences between model systems (in which the majority of cells are isogenic) and cancer samples (which have high levels of chromosomal and gene mutation heterogeneity) are reflected by the display of mainly micro-evolutionary processes in model systems, and a mixture of macro-evolution plus micro-evolution in real cancer. In a sense, many model systems mimic a population of the same species, while real cancer systems mimic a population of the same species and different species [4, 63–65].

Fourth, when discussing the advantages/disadvantages of aneuploidy, the majority of studies are focused on growth status. It should be pointed out that while growth represents a key feature of cancer, during the earlier stages of cancer evolution, growth might not necessarily be the key precondition. The rationale of focusing on cell proliferation in cancer research was based on the concept of accumulating gene mutations during cancer initiation and progression; it was thus argued that the proliferated cell population could provide the basis for stepwise cancer evolution. Since the discovery that punctuated cancer evolution is achieved by genome reorganization events, such as genome chaos, the rationale of focusing on proliferation has been challenged [6–8, 50, 56, 66, 67]. Surely, the cancer genome sequencing project has failed to detect serial, stepwise gene mutation accumulation in the majority of cancer cases [4, 59, 68]. In contrast, system instability might not only be an important earlier event, but in fact the key event. According to the genome theory [4, 49, 50, 56], genome instability could be the key driver for all major transitions for cancer evolution, including transformation, metastasis, and drug resistance. It is likely that cellular proliferation contributed by the “oncogenes” often represents the later events which help cancer cells to become more dominant cell populations (for more, see reference [4, 37]). Similar patterns have been observed in metastasis and drug resistance. Therefore, system instability might be the most important aspect for the success of cancer: new systems’ emergence from normal tissue [69, 70]. Recent single-cell sequencing of breast cancer cells supports this viewpoint. It was observed that copy number changes and rearrangements appeared early in tumorigenesis. In contrast, point mutations occurred gradually during tumor evolution (within the micro-evolutionary phase) [71].

Fifth, most current research efforts are focusing on molecular profiles based on an average population, and outliers are eliminated or ignored, either by the methods used or statistical tools. The traditional view of biological research is to identify patterns from “noise,” without the realization that the so-called “noise” in fact is heterogeneity, which represents a key feature of cancer evolution by functioning as the evolutionary potential. Increased studies have demonstrated the importance of outliers in cancer evolution, as cancer is an evolutionary game of outliers [4, 72, 73].

Sixth, in the search for the molecular consequence of aneuploidy, the focus is still on the genes’ function. Despite the fact that it is hard to make sense out of the data of altered profiles of a large numbers of genes, few have realized that aneuploidy, in fact, changes a new chromosomal-level coding system, which is namely the system inheritance [16, 37, 66].

Clearly, a new framework is needed to systematically study aneuploidy in cancer evolution. Since cancer is a complex adaptive system, and each run of successful evolution can be linked to different genome and gene mutation profiles, more attention needs to be paid to the gap between initial conditions and final emergence, the environmental and genome contexts, landscape dynamics, and system instability-mediated cancer evolutionary potential [59]. Because cancer evolution requires inheritance, and involves the emergence of new systems, the following session will focus on these issues to redefine inheritance and the emergent bio-cellular system.

### The genome theory of cancer evolution

Based on the ultimate importance of chromosomal aberrations in cancer evolution, especially within the punctuated phase of macro-cellular evolution, the genome theory of cancer evolution was introduced with the aim of departing from the gene mutation theory of cancer [4, 49, 66]. To illustrate how chromosomal changes play a key driving role in cancer evolution, we have redefined the genomic meaning of karyotype changes, and compared the evolutionary dynamics between clonal and non-clonal chromosomal aberrations [6–8, 64, 74]. Moreover, we have proposed the use of the genome-mediated evolutionary mechanism to unify the diverse molecular mechanisms of cancer [55, 75]. Since aneuploidy represents one important type of karyotype aberration [15, 74], the principles of genome theory can be easily applied to aneuploidy research in the context of somatic evolution, complexity, and how chromosomally-defined new genomic information plays a driving role for new system emergence.

#### System inheritance and aneuploidy

Genes encode proteins, and the sequence of ATGC within genes is the genetic coding. It has been challenging to study how aneuploidy affects genetic coding when there are over a thousand genes involved. Traditionally, attention has been paid to dosage effects. With the development of the technical platform for transcriptome profiling, it was surprisingly observed that the impact of aneuploidy is far beyond the dosage effect on genes located on gained or lost chromosomes [40, 76, 77]. Even more interestingly, different experimental systems differ in terms of the observed impact. The genomic basis for these unexpected findings is unknown.

During our watching-evolution-in-action experiments within an in vitro immortalization model, we constantly observed rapid and massive genome re-organization during the punctuated phase of cancer evolution [4, 6–8, 78]. Remarkably, during this phase, mother cells can generate daughter cells with similar DNA but drastically different karyotypes. To illustrate the biological meaning of this karyotype re-organization, we realized that the shattering of the genome and its subsequent reorganization represent a powerful means of creating new genomic information. Such a new mechanism functions above the coding of individual genes, and perhaps serves to organize gene interaction.

One of the biggest promises of the human genome sequencing project was to decipher the blueprint that makes us human. Unfortunately, we have failed to achieve this goal following the sequencing phase of the genome project. Despite that we know the sequence of nearly all genes, we have no idea what the genomic blueprint is. Systems biologists have suggested that the network structure defines the blueprint. But what defines the network structure in the first place?

Putting all of these questions together, we realized that the karyotype, in fact, represents a new genomic coding system, and the blueprint is encoded by the new genomic information which is defined by the order of genes along and among chromosomes [4, 37, 59]. More specifically, a gene only encodes a specific “part inheritance,” while a set of the chromosomes of a given species encodes the “system inheritance” [16, 66]. Furthermore, we suggested that the karyotype defines the boundary of a network structure for a given species, which integrates the network into the genome-defined system [69, 70].

Further studies suggested that karyotype coding is maintained by the function of sex through the meiotic pairing mechanisms [79–82]. Nearly all significant karyotype aberrations will be eliminated by the “reproductive filter,” which ensures the species identity. In this way, similar gene content can form different species by creating different karyotypes, which determine the physical platform for gene interactions in the 3-D nucleus [37]. Since different species display different karyotypes, a species is in fact preserved by its own chromosomal coding. Furthermore, it is likely that altered genomic information contributes to many common and complex diseases [4, 37].

Obviously, aneuploidy alters the karyotype and thus changes the genomic coding. Despite the fact that much work is needed to illustrate the details of how aneuploidy changes genomic coding, many experiments support this idea in principle. For example, aneuploidy not only changes the overall transcriptomes, but can specifically provide new functions to rescue cells lacking specific essential genes. When the only copy of the MYO 1 gene was knocked out, yeast should no longer have been able to survive, as MYO1 encodes the myosin II protein required for cytokinesis. Surprisingly, however, extensive polyploidy and aneuploidy (rather than reverse mutation) was demonstrated to rescue the dying populations, illustrating that genome-level changes can generate emergent new phenotypes without directly fixing the specific deleted gene [83]. In other words, re-organizing the karyotype coding can create functions encoded by specific genes in different systems. Ample evidence can be found in current literature [4, 37].

#### Fuzzy inheritance and aneuploidy

One key feature of cancer is its multiple levels of genetic/epigenetic/genomic heterogeneity. During time-course experiments designed to trace karyotype evolution in vitro, it was documented that the degree of karyotype heterogeneity can be drastically different depending on the phases of cellular evolution [6–8]. In addition, the different extents of karyotype heterogeneity are evolutionary phase-specific (extremely high within the punctuated phase and low within the stepwise phase), suggesting that karyotype heterogeneity is inheritable among different cell populations. A similar phenomenon has been observed from DNA mutation when discussing the mutant type [84]. Recently, the two phases of cancer evolution have been confirmed by gene mutation and copy number profiling [71, 85–88].

Following the characterization of various cancer cell lines, it became clear that each line displays a different degree of heterogeneity (reflected as the rate of NCCAs). To establish the baseline of karyotype heterogeneity in normal individuals, SKY karyotype analysis was used after short-term culture of lymphocytes, and the rate of structural NCCAs was found to be around 1–4%. Interestingly, drug treatment-induced frequencies of NCCAs are also different among cell lines or individuals with different levels of genome instability, and elevated frequencies of NCCAs from lymphocytes are detected from various diseases or illness conditions [17, 19, 89].

The above observations are highly significant in the context of missing inheritability [90, 91]. It is generally accepted that phenotype is the result of the interaction of genotype and environment, but its mechanism is not clearly understood. For example, for phenotype plasticity, the mechanism is unknown. It is also unclear how different genotypes display different extents of phenotypic plasticity, and why environment can win over the power of genetics or vice versa.

The link between the frequency of NCCAs and phenotype heterogeneity has promoted the concept that the previously regarded “noise,” in fact, represents karyotype heterogeneity. Further research/synthesis has led to the realization that it is likely that the coded message at the karyotype level is heterogeneous in nature, which results in high phenotypic plasticity.

Important questions were then asked. Is it possible that inheritance itself is not precise but fuzzy, even for the coding of a single gene-determined phenotype? Do genetic elements code a spectrum of potential information rather than a fixed one? What if these highly penetrant relationships between genotype and phenotype only represent exceptions in which environmental factors are well-controlled? Does the major role of environmental factors select a specific possibility encoded by the genetic coding? Do stress conditions increase the heterogeneity of phenotype by increasing the fuzziness of the genetic coding? To address these questions, fuzzy inheritance has been introduced by us as the mechanism of various levels of genetic and epigenetic heterogeneity [4, 37, 70].

Since non-clonal aneuploidy belongs within the category of NCCAs and represents karyotype heterogeneity, it is important to integrate aneuploidy into fuzzy inheritance. Despite the fact that the frequencies of aneuploidy in various normal tissues are low, when combined with other NCCAs, the level of altered karyotypes is rather high, especially under stress conditions [50, 92, 93, 94]. In addition, the drastic difference between the spontaneous rate of aneuploidy in cells from normal tissue and in those from cancers supports the idea that specific cell populations display different degrees of fuzzy inheritance which are related to aneuploidy. For example, the mis-segregation rate in a stable, diploid cell line is one chromosome per 100–1000 cell divisions. In contrast, the mis-segregation rate in cultured cancer cells with CIN is approximately once every 1–5 divisions [95–97]. More remarkably, during the genome chaos phase, almost all cells display a high rate of mis-segregation with large number of aneuploidies, plus all sorts of karyotype variants [6, 50, 67]. The high degree of fuzzy inheritance in cancer, in fact, can also explain why non-clonal aneuploidy is a common feature of even later stages of cancer. All tumors are under high stress from surrounding tissues or higher systems, so fuzzy inheritance-mediated karyotype heterogeneity is essential for tumor survival and further progression. Clearly, how aneuploidy quantitatively contributes to fuzzy inheritance-mediated genomic heterogeneity needs further study.

#### The relationship between cellular adaptation and trade-off

Traditionally, aneuploidy has long been blamed as the result of bio-errors. Most of the molecular evidence supports this viewpoint, as when specific genes are dysfunctional as a result of experimental manipulation, a phenotype of increased aneuploidy can be observed. Many gene mutations involving cell cycle/chromosomal integrity can achieve the same phenotype. While the baseline of aneuploidy in normal individual tissues is low in many cases, in some tissue types, the spontaneous aneuploidy is high. Moreover, the overall rate of NCCAs is not low at all in most normal tissues.

Obviously, the higher than expected frequency of karyotype changes, including aneuploidy, cannot simply be explained as bio-error. In recent years, the biological significance of these seemingly random genetic “backgrounds” were studied, which has led to the appreciation of genomic heterogeneity in cancer evolution. Further synthesis suggests a relationship between stress-induced NCCAs and the advantages offered by their presence for cellular adaptation, as well as the trade-offs caused by their presence in cancer evolution and possibly in other disease conditions [4, 92]. Moreover, many diseases are the results of genomic variants which do not fit the current environments. Due to the dynamics of environments and the nature of fuzzy inheritance, it is impossible to eliminate all of these variants. Paradoxically, these genomic variants might be necessary for the species’ long-term survival, and they should be considered as a life insurance policy despite their high costs. Such a concept of trade-off not only addresses the key evolutionary mechanism of many diseases including cancer, but may also provide some answers to patients who ask the “why me” question. In a sense, cancer as an evolutionary trade-off can be illustrated by different perspectives: at the mechanistic level, cancers are the by-products of evolution (that is, the same mechanisms which make us human also make cancer successful); at the species level, as population heterogeneity is important for species survival, an individual with high genome instability can be considered as paying the price for our species; and at the individual level, most bio-features, including lifestyle, could be beneficial in some aspects and yet harmful in other aspects. Even for non-clonal aneuploidy-mediated cellular heterogeneity, while this phenomenon can provide a potential advantage for cellular adaptation, it can also, paradoxically, generate non-specific system stress, which can further produce more genetic and non-genetic variants which favor the disease condition [4]. Based on this rationale, we have attempted to use type I and type II CIN to unify diverse gene mutations under the principle of CIN-mediated cancer evolution, as many gene mutations and molecular pathways which are not directly involved in maintaining genome integrity can still be linked to CIN [50].

#### Emergence and luck

The unpredictability of emergence represents a common challenge for using parts characterization to predict phenotype at higher levels in a complex adaptive system. How aneuploidy triggers the successful evolution of cancer, especially during the phase transitions, is almost unknown. The situation worsens when the type of aneuploidy is non-clonal, and when both the context of other types of genetic changes and cellular environments keeps changing. For example, different tissues can tolerate different degrees of aneuploidy; aneuploidy can be detected at the early development stage with high frequencies, but the developmental process can overcome them, whereas the impact of aneuploidy can become serious during cancer evolution later in life; even in tissues that are sensitive to aneuploidy, most instances of aneuploidy will not lead to cancer. It seems that in different tissue types, different stages of development and aging, and different physiological and pathological processes, there are different “roles” for the cellular society which favor different types of emergence [19, 37]. For example, in a normal physiological cellular society, the average profile can overrule outliers, while in the setting of cancer evolution and under high stress, the outliers may triumph.

**Fig. 1:**
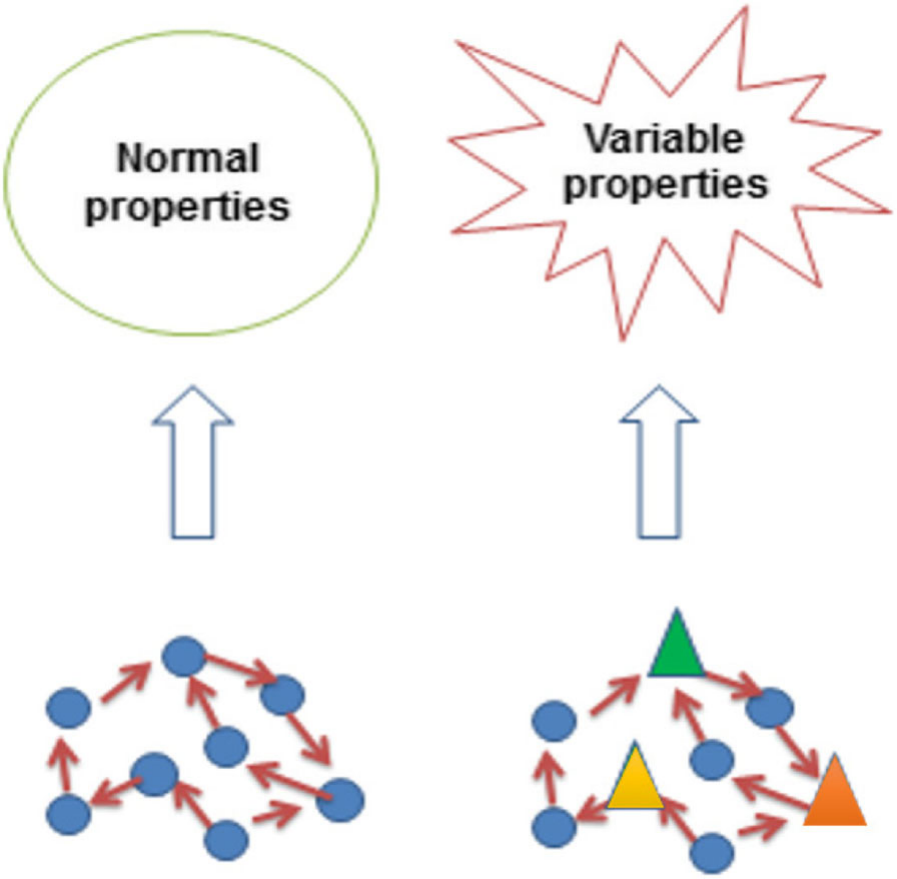
The illustration of how the heterogeneity of aneuploidy impacts the emergent properties of cellular populations. Since there is no direct correlation from individual agents to the emergent properties, the final properties are based on the collective emergence of all agents. Circles represent cells with normal karyotypes, triangles represent cells with non-clonal aneuploidy, and arrows represent pathways among agents. These variable properties are the potential basis for cancer evolution (modified from reference [19])

To understand how non-clonal karyotype aberrations can contribute to the emergence of cancer evolution, we proposed that instances of non-clonal aneuploidy, like other types of non-clonal karyotype aberrations, serve as heterogeneous agents that can impact the emergent properties of the cellular evolution. While the details of how aneuploidy affects emergence are not yet known, this model illustrates the importance of how even a portion of non-clonal aneuploidy can change the emergence process (Fig. 1). A similar general model of how the heterogeneity of genetic agents impacts diseases has been proposed to explain how NCCAs can contribute to different diseases [18, 98].

Due to the complex combinations of aneuploidy and the genetic and environmental contexts, a vast majority of these combinations will not directly lead to cancer’s success, as they are either not powerful enough to contribute to the phase transition which leads to cancer, or they are eliminated by system constraint. For example, it was recently demonstrated that the complex karyotypes derived from aneuploidy can trigger the immune system to eliminate them (Table 3). Another example is drug therapy in which a high dosage of drugs is used. The majority of the cancer cells will be eliminated by the initial drug treatment, and only a tiny portion of the cancer cells can survive (through the formation of genome chaos). It is extremely challenging to predict which aberrations will be successful, even though drug-resistant clones often arise.

As a consequence of the highly heterogeneous nature of karyotypes featuring aneuploidy, as well as the diverse genomic/environmental contexts involved, most genomic aberrations will not lead to the success of cancer, despite their potential. A “perfect storm” is needed for any cancer to be successful. Under such conditions, luckiness or unluckiness can be considered agents which impact the emergent properties.

**Fig. 2:**
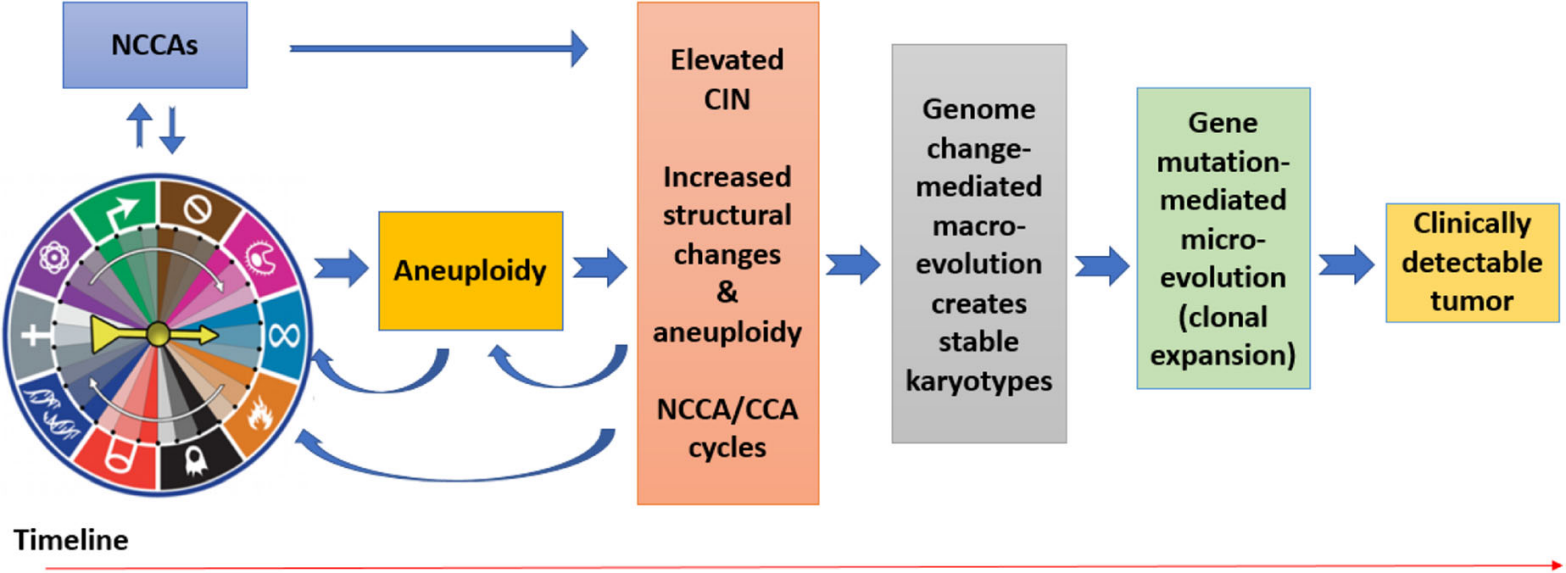
The proposed timeline that illustrates the relationship between various molecular mechanisms (summarized by the hallmarks of cancer, modified from reference [50, 139]), aneuploidy, CIN (often coupled with other karyotype alterations such as structural alterations and polyploidy), macro-evolution, micro-evolution and the clinically detectable tumor. As NCCAs can be detected from earlier developmental stages, the relationship between various molecular mechanisms and aneuploidy is less clear. It is clear, however, that there is a complex, interactive relationship. Furthermore, elevated CIN is important for triggering macro-cellular evolution, followed by micro-cellular evolution, leading ultimately to the proliferation of the cancer cells with the winning genome. This diagram highlights the complex, dynamic relationship between aneuploidy, CIN and the two phases (macro and micro) of cancer evolution

Such interplay during cancer evolution is ultimately responsible for the emergence of a new genome system from normal tissue, and aneuploidy-mediated genome re-organization plays a key role for creating these new systems [4, 37, 66]. For altered cells to become cancer cells, they have to complete many key transitions, including immortalization, transformation and metastasis, all of which require the emergence of different genome systems; gene mutation alone is not sufficient for creating a new system. The alteration of system inheritance and the increased degree of fuzzy inheritance mainly contribute to the macro-cellular evolution which leads to new systems. In contrast, genes that promote cell proliferation can expand cancer cell populations following the formation and selection of cancer cells with unique karyotype-defined systems (Fig. 2).

## Conclusion and future research

Within the framework that represents cancer as a complex adaptive system, the following elements become highly important for understanding both the key feature and common mechanism of cancer: internal and external stress-mediated adaptation and its trade-off (trigger factors); the multiple levels of genetic/environmental heterogeneity (essential conditions for cancer evolution); the involvement of system inheritance and fuzzy inheritance (how genomics works during cancer evolution); the two phases of cancer evolution (the mechanism of cellular evolution and the relationship between gene/epigene and genome changes); the emergence of new, karyotype-defined systems (the formation of a cancer seed and the importance of NCCAs and outliers); and the population of cancer cells that become clinically significant (the dominance of cancer). It is necessary to integrate these elements during studies of aneuploidy.

Despite the recent exciting progress of aneuploidy research, some great challenges remain. Simply focusing on the molecular characterization of agents at lower levels is neither sufficient for understanding the emergent properties of a complex adaptive system nor for predicting the contribution of aneuploidy to cellular evolution.

To change the status quo, the crucial first step is to acknowledge the fundamental limitation of the reductionist approach in aneuploidy research, as there is no precise, predictable relationship between an understanding of the individual mechanism of aneuploidy and clinical certainty, nor between many diverse individual agents and the emergent properties of cancer evolution. It is thus equally difficult to search for patterns based on diverse molecular pathways. In addition, the dynamic interaction of average cells and outliers further complicates this prediction. To make sense of this complexity and to increase predictability, a better strategy is to consider aneuploidy as an agent and cancer as a complex adaptive system. Expectations regarding the predictive power of aneuploidy must also change, as the success of cancer evolution depends on both evolutionary potential (which can be measured) as well as on chance or accidents (which are hard to predict) [99, 100]. The importance of special “circumstances” or “accidents” in evolutionary success is receiving our increased attention [4, 37, 66].

An innovative type of biomarkers is needed to integrate aneuploidy with other karyotype alterations, and these should be used to measure evolutionary potential (based on the degree of heterogeneity and karyotype complexity) rather than specific pathways. This approach will likely bridge the gap between basic research and clinical implications. There are some examples of applying aneuploidy in clinical analysis [101]. High somatic copy number alterations in melanoma patients have recently been linked with less effective response to immune checkpoint blockade anti–CTLA-4 (cytotoxic T lymphocyte–associated protein 4) therapy [52]. Clearly, aneuploidy status is associated with response to precise immunotherapy. We have engaged in the effort of using NCCAs (mainly structural NCCAs) to monitor clinical outcomes. One approach is to measure an individual’s overall genome instability and its linkage to cancer status. We have observed a strong correlation between the frequencies of structural NCCAs from short term lymphocytes culture and prostate cancer [37]. This work has expanded into other health conditions [17]. A similar concept of monitoring overall genome instability to detect cancer can be found in literature involving telomere length and the overall chromosomal aberration rate [102–107]. More aneuploidy data should be integrated into this effort. In particular, since chromosome data (CIN statuses, for example) have much more clinical predictive power than sequenced gene mutation data [4, 50–53, 74], bioinformaticians should be encouraged to search for new platforms for mining sequences in the context of evolutionary potential, through the use of AI (artificial intelligence) approaches. For example, this strategy could be used to search for the principle of how aneuploidy changes the blueprint, its overall impact on the gene network, and the quantitative contribution of elements for the higher level of emergence.

Further research is also needed to compare emergence based on average profiles and outliers with various degrees of system stress. Such analysis needs to be done within the context of the cellular society concept [4, 108]. As for the technical platforms, new monitoring methods should be developed to study single cells, especially to profile non-dividing cell populations. Recently, the CRISPR/Cas9 system has been used to eliminate targeted chromosomes. This new approach offers an effective way to develop animal models with aneuploidy, which can be used as a potential therapeutic strategy for human aneuploidy diseases [109]. Certainly, among these advances, one immediate priority is to illustrate how aneuploidy triggers structural alterations of the karyotype and provides the maximal diversity and plasticity needed for new system emergence and domination. For example, can aneuploidy lead to genome chaos [110]? How does the heterogeneity of aneuploidy impact the newly emergent karyotype?

Finally, and perhaps most importantly, the ultimate goal of establishing better concepts and platforms for cancer research is to apply them in the clinic. Further studies are needed to apply this new understanding of aneuploidy for patient stratification, directing therapy schedules, and predicting drug resistance.

## Abbreviations

CCA: clonal chromosome aberrations
C-Frags: chromosome fragmentations
CIN: chromosomal instability
CNV: copy number variations
DMFs: defective mitotic figures
NCCA: non clonal chromosome aberrations
